# Appropriate use criteria for optical coherence tomography guidance in percutaneous coronary interventions: Recommendations of the working group of interventional cardiology of the Netherlands Society of Cardiology

**DOI:** 10.1007/s12471-018-1143-z

**Published:** 2018-08-31

**Authors:** A. J. J. IJsselmuiden, E. M. Zwaan, R. M. Oemrawsingh, M. J. Bom, F. J. W. M. Dankers, M. J. de Boer, C. Camaro, R. J. M. van Geuns, J. Daemen, D. J. van der Heijden, J. W. Jukema, A. O. Kraaijeveld, M. Meuwissen, B. E. Schölzel, G. Pundziute, P. van der Harst, J. van Ramshorst, M. T. Dirksen, C. Zivelonghi, P. Agostoni, J. A. S. van der Heyden, J. J. Wykrzykowska, M. J. Scholte, H. M. Nef, M. J. M. Kofflard, N. van Royen, M. Alings, E. Kedhi

**Affiliations:** 1 Department of Cardiology, Amphia Hospital, Breda, The Netherlands; 2 Department of Cardiology, Albert Schweitzer Hospital, Dordrecht, The Netherlands; 3 Department of Cardiology, Erasmus Medical Centre, Rotterdam, The Netherlands; 4 Department of Cardiology, VU Medical Centre, Amsterdam, The Netherlands; 5 Department of Radiation Oncology, GROW, School for Oncology and Developmental Biology, Maastricht University Medical Centre, Maastricht, The Netherlands; 6 Department of Radiation Oncology, Radboud University Medical Centre, Nijmegen, The Netherlands; 7 Department of Cardiology, Haaglanden Medical Centre, the Hague, The Netherlands; 8 Department of Cardiology, Leiden University Medical Centre, Leiden, The Netherlands; 9 Department of Cardiology, University Medical Centre Utrecht, Utrecht, The Netherlands; 10 Department of Cardiology, University Medical Centre Groningen, Groningen, The Netherlands; 11 Department of Cardiology, Northwest Clinics, Alkmaar, The Netherlands; 12 Department of Cardiology, St Antonius Hospital, Nieuwegein, The Netherlands; 13 Department of Cardiology, Academic Medical Centre, Amsterdam, The Netherlands; 14 Department of Cardiology, University Hospital of Giessen and Marburg, Standort Giessen, Giessen, Germany; 15 Department of Cardiology, Isala Clinics, Zwolle, The Netherlands; 16 Department of Cardiology, Radboud University Medical Centre, Nijmegen, The Netherlands

**Keywords:** Coronary artery disease, PCI, OCT

## Abstract

**Introduction:**

Optical coherence tomography (OCT) enables detailed imaging of the coronary wall, lumen and intracoronary implanted devices. Responding to the lack of specific appropriate use criteria (AUC) for this technique, we conducted a literature review and a procedure for appropriate use criteria.

**Methods:**

Twenty-one of all 184 members of the Dutch Working Group on Interventional Cardiology agreed to evaluate 49 pre-specified cases. During a meeting, factual indications were established whereupon members individually rated indications on a 9-point scale, with the opportunity to substantiate their scoring.

**Results:**

Twenty-six indications were rated ‘Appropriate’, eighteen indications ‘May be appropriate’, and five ‘Rarely appropriate’. Use of OCT was unanimously considered ‘Appropriate’ in stent thrombosis, and ‘Appropriate’ for guidance in PCI, especially in distal left main coronary artery and proximal left anterior descending coronary artery, unexplained angiographic abnormalities, and use of bioresorbable vascular scaffold (BVS). OCT was considered ‘Rarely Appropriate’ on top of fractional flow reserve (FFR) for treatment indication, assessment of strut coverage, bypass anastomoses or assessment of proximal left main coronary artery.

**Conclusions:**

The use of OCT in stent thrombosis is unanimously considered ‘Appropriate’ by these experts. Varying degrees of consensus exists on the appropriate use of OCT in other settings.

**Electronic supplementary material:**

The online version of this article (10.1007/s12471-018-1143-z) contains supplementary material, which is available to authorized users.

## Introduction

Intracoronary optical coherence tomography (OCT) is a catheter-based, high-resolution imaging technique using backscattering of near-infrared light for the characterisation of the coronary artery wall, plaque morphology/pathology and intracoronary devices such as stents [1–3].

Compared with intravascular ultrasound (IVUS), OCT images are acquired faster and the axial resolution is higher [1–3]. IVUS has a larger penetration depth of 4–8 mm versus 0.1–2 mm for OCT, depending on the variable attenuation of near infrared light by various tissue types [1–3]. Because of its detail in visualisation of plaque composition, dissection, thrombus and stents, OCT is increasingly used during coronary angiography and PCI.

The wealth of information that is gained with this technique needs to be placed in a clinical perspective. Although several expert groups have formulated standards on acquisition, measurements, terminology and clinical applications of OCT [1–3], evaluation according to an analysis of appropriate use criteria in combination with a literature review is lacking. This document aims to provide a framework for the appropriate use of OCT in daily clinical practice.

## Methods

This document covers a range of scenarios representing everyday clinical practice.

Twenty-one of all 184 members of the Dutch Working Group on Interventional Cardiology responded positively to creating a consensus panel (Tab. 1), forming a representative reflection of the thirty Dutch cardiovascular intervention clinics. Panellists were asked to submit clinical scenarios as encountered within their own practice, according to a fixed format (Tab. 2). Scenarios were categorised by indication, and reviewed until consensus on factual indications was reached.

**Table 1:** Formation Dutch working group on optical coherence tomography

Number of delegates/centre (*N* = 21)	Participating hospital
1	Amsterdam Medical Centre, Amsterdam
3	Amphia Hospital, Breda
1	Albert Schweitzer Hospital, Dordrecht
2	Erasmus Medical Centre, Rotterdam
1	Leiden University Medical Centre, Leiden
1	MC Haaglanden, the Hague
2	Northwest Clinics; Alkmaar
3	Radboud University Medical Centre, Nijmegen
3	St Antonius Hospital, Nieuwegein
2	University Medical Centre Groningen, Groningen
1	University Medical Centre Utrecht, Utrecht
1	VU University Medical Centre, Amsterdam

**Table 2:** Fixed format of clinical scenarios^a^

1	Clinical presentation
2	Risk factors and comorbidities
3	Cardiac history
4	Non-invasive tests results to evaluate the presence and severity of myocardial ischaemia; electrocardiography, laboratory and non-invasive ischaemia detection
5	Formal coronary angiography reports
6	Invasive testing such as intravascular ultrasound and fractional flow reserve

^a^All submitted scenarios for the use of optical coherence tomography were developed according to a fixed format considering all the above mentioned common variables

### Appropriate use criteria

Use of OCT was defined appropriate when ‘potential benefits, in terms of health outcomes (survival, symptoms, functional status, and/or quality of life), exceed negative consequences of the treatment strategy’ [4].

Scenarios were scored on a 1–9 scale:

*Scores 7–9:* Appropriate; OCT likely improves health outcomes.

*Scores 4–6:* May be appropriate; Uncertainty that OCT improves health outcomes.

*Scores 1–3:* Rarely appropriate; Unlikely that OCT improves health outcomes.

According to prescribed assumptions (Tab. 3; [5]), panellists anonymously scored scenarios.

**Table 3:** General assumptions^a^

1	Operators performing percutaneous revascularisation have appropriate clinical training, experience and have satisfactory outcomes as assessed by quality assurance monitoring
2	Revascularisation is performed according to international established standards of care [5]
3	The rating panel should rate the appropriateness of the use of OCT on the basis of the clinical scenario presented, including the observed coronary disease, independently of a judgment about the appropriateness of the coronary angiogram within the given scenario
4	There are no other significant coronary artery stenoses present apart from those described in the clinical scenario
5	Significant coronary stenosis in the clinical scenarios is defined as ≥70% luminal diameter narrowing on angiography or intermediate angiographic luminal narrowing (40–70%), with an abnormal FFR
6	FFR ≤0.80 is abnormal and is consistent with downstream ischemia
7	Clinical stent strut malapposition is defined as ≥1–2 mm distance between the stent strut and the intimal surface in more than 5% of the total surface area of the stent

*FFR* fractional flow reserve, *OCT* optical coherence tomography

^a^To limit inconsistencies in interpretation, these specific assumptions were considered when interpreting the ratings

### Statistics

Scores were categorised according to the appropriate use criteria scale [4]. Overall scores of each indication for OCT were described descriptively as mean ± SD. Outliers were defined as observations 1.5 times the interquartile range above the third quartile or below the first quartile.

## Results

Twenty-one cardiologists rated 49 submitted OCT scenarios (Tab. 4). Anonymised individual scores are registered online; Fig. 1, supplemental Appendix Figs. 2–14.

**Table 4:** Summary of clinical scenarios with corresponding ratings

Case	Indication (corresponding appendix)	Appropriate use rating	SD
*Identification of culprit lesion in acute coronary syndrome *(Fig. 1)			
1	Identification culprit lesion in NSTEMI with angiographic two significant stenosis and no decisive answer on which one is the culprit	M (6)	±1.37
2	Identification mechanism STEMI (spasm vs. plaque rupture) after thrombectomy followed by severe spasm	M (5)	±2.41
3	Identification culprit lesion in NSTEMI with abnormal ECG and angiographically no evident thrombus or occlusion	A (7)	±1.77
4	Identification plaque erosion	A (7)	±2.02
5	Identification culprit lesion in OHCA with angiographic signs (haziness)	A (7)	±2.36
6	Identification culprit lesion in MI with abnormal ECG and angiographically intermediate stenosis	M (5)	±1.88
*Evaluation of stent thrombosis* (supplemental Appendix Fig. 2)			
7	Identification of stent thrombosis mechanism in a STEMI patient	A (8)	±0.68
8	Re-evaluation with OCT after STEMI of a hazy non-culprit lesion which was initially treated conservatively	M (6)	±1.94
9	Evaluation of mechanism in recurrent STEMI due to stent thrombosis in proximal LAD	A (9)	±0.69
*Evaluation of strut coverage *(supplemental Appendix Fig. 3)			
10	Evaluation of strut coverage 4 weeks after initial stent placement in a patient with high bleeding risk (discontinuing DAPT)	M (4)	±2.51
11	Evaluation of strut coverage 12 weeks after initial stent placement in a patient with high bleeding risk who requires surgery (discontinuing DAPT)	R (3)	±1.98
12	Evaluation of BVS after ~1.5 years for discontinuing DAPT	M (4)	±2.76
*OCT-guided PCI in critical lesions *(supplemental Appendix Fig. 4)			
13	Guiding in complicated PCI with unknown apposition/position of the stent in the LMCA and post PCI with possible stent fracture after overexpansion	A (8)	±1.26
14	Guiding in PCI with bifurcation lesion for sizing and stent strategy	M (5)	±2.21
15	Guiding in PCI to determine landing zone stent and stent length in angiographically diffuse long lesion	M (6)	±2.00
16	OCT next to significant FFR for evaluation stenosis severity	R (2)	±1.83
17	OCT next to non-significant FFR for evaluation stenosis severity	R (3)	±1.74
*OCT guidance in PCI in LMCA *(supplemental Appendix Fig. 5)			
18	OCT guidance in PCI of the proximal LMCA	R (3)	±1.46
19	OCT guidance in PCI of the distal LMCA	A (7)	±1.66
*Evaluation of stent apposition *(supplemental Appendix Fig. 6)			
20	Evaluating thrombosis mechanism in extensive stent thrombosis	A (9)	±1.03
21	Evaluating stent apposition post PCI in non-complex lesion	M (4)	±2.39
22	Evaluating severe calcified lesion for treatment strategy (rotablator?)	R (3)	±1.56
23	Evaluating stent apposition after rotablator treatment in complex diffuse long lesion and placement of multiple stents	A (7)	±1.85
24	Evaluating stent apposition after extensive post-dilatation in an initially undersized stent	A (7)	±2.22
*Identification of unexplained angiographic abnormalities *(supplemental Appendix Fig. 7)			
25	Unravel the mechanism for distal occlusion in coronary artery without proximal lesion (local problem or emboli with other origin?)	A (7)	±2.48
26	Control OCT 5 days after initial angiography in NSTEMI patient which was treated conservatively	A (7)	±1.39
27	Evaluation haziness (thrombus) in proximal LAD in STEMI patient with incurable cancer (local problem or emboli?)	A (7)	±2.17
28	Discrepancy between angiographic finding (intermediate stenosis) and FFR (borderline significant)	M (6)	±2.28
29	Evaluation angiographic haziness in transient STEMI	A (7)	±1.60
*Identification of dissection *(supplemental Appendix Fig. 8)			
30	Confirmation of SCAD in young patient without classical risk factors for atherosclerotic coronary artery disease	M (6)	±2.09
31	Identification thrombosis mechanism after thrombosuction resulting in a normal angiography in a patient with a mechanical valve	A (7)	±2.16
32	Confirmation of SCAD in young patient with classic risk factors for atherosclerotic coronary disease	M (6)	±2.24
*Stent sizing *(supplemental Appendix Fig. 9)			
33	Sizing for covered stent with risk on blocking substantial side branch	A (7)	±2.55
34	Sizing for stent in hazy angiography with multiple complex lesions	M (6)	±1.80
35	Stent sizing in bifurcation lesion (pre PCI)	M (6)	±1.94
*Evaluation of stent apposition in critical lesions *(supplemental Appendix Fig. 10)			
36	Control OCT after 2 weeks to evaluate stent apposition in proximal LAD with suspected malapposition during initial angiography	A (6.5)	±2.22
37	Control OCT for stent apposition in a patient with high bleeding risk and angiographically suspected under-expansion	M (6)	±1.97
38	Evaluating stent apposition in bifurcation lesion (post PCI)	A (7)	±1.53
39	Identification of the mechanism behind a distal occlusion in a coronary vessel with multiple mild plaques proximally (local or emboli of other origin?)	A (7)	±1.35
40	Evaluating stent apposition in a patient with high bleeding risk with the intention to keep the duration of DAPT treatment as short as possible	A (7)	±1.81
41	Routine use of OCT for evaluation stent apposition in PCI of proximal LAD	M (4)	±1.88
*In-stent restenosis *(supplemental Appendix Fig. 11)			
42	OCT identification of the mechanism of ISR in order to guide therapy, i. e. DES vs. DEB after 1st restenosis	M (6)	±1.64
43	OCT identification of the mechanism of ISR in order to guide therapy, i. e. DES vs. DEB after 2nd restenosis	A (7)	±2.35
44	OCT identification of the mechanism of ISR in order to guide therapy, i. e. DES vs. DEB after 3rd restenosis	A (7)	±1.58
*Implantation dedicated stent *(supplemental Appendix Fig. 12)			
45	Evaluation of stent apposition in a BVS	A (8)	±2.10
46	Evaluation of stent apposition in a self-expandable stent	A (7)	±2.16
*OCT in grafts *(supplemental Appendix Fig. 13)			
47	Detection of early cardiac allograft vasculopathy after heart transplant	M (4)	±2.45
48	Detection of stenosis of a CABG anastomosis	R (3)	±1.87
*OCT in CTO *(supplemental Appendix Fig. 14)			
49	Evaluation of multiple dissection-like images outside the stent in the sub-intimal path of a previous CTO during follow-up angiography after CTO recanalisation	A (7)	±2.48

The number in parentheses next to the rating reflects the rounded off mean score for that indication.

*A* appropriate care, *BVS* bioresorbable vascular scaffold, *CTO* chronic total occlusion, *DAPT* dual antiplatelet therapy, *DEB* drug-eluting balloon, *DES* drug-eluting stent, *ECG* electrocardiogram, *FFR* fractional flow reserve, *ISR* in-stent restenosis, *LAD* left anterior descending coronary artery, *LMCA* left main coronary artery, *M* may be appropriate care, *MI* myocardial infarction, *NSTEMI* non-ST-elevated myocardial infarction, *OCT* optical coherence tomography, *OHCA* out-of-hospital cardiac arrest, *PCI* percutaneous coronary intervention, *SCAD* spontaneous coronary artery dissection, *SD* standard deviation, *STEMI* ST-elevated myocardial infarction, *R* rarely appropriate, *RCA* right coronary artery

**Fig. 1:**
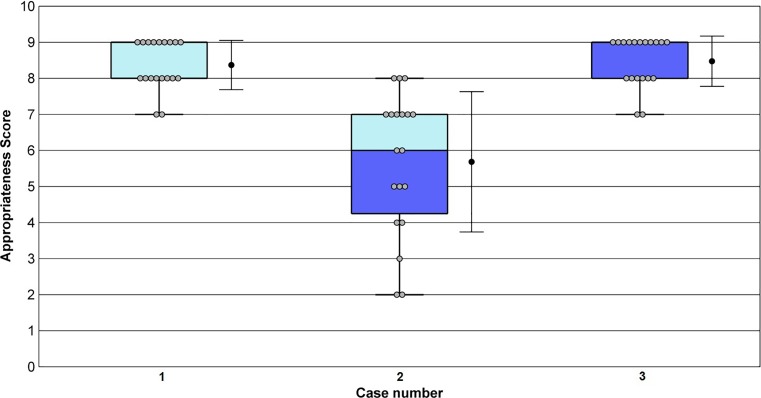
OCT appropriate use criteria scores for evaluation of stent thrombosis. On each *box*, the central mark indicates the median, and the bottom and top edges of the box indicate the 25th and 75th percentiles, respectively. The *whiskers* extend to the most extreme data points not considered outliers, and the outliers are plotted individually as a *red dot*. The *grey dots* represent the individual scores of the panellists. The *whiskers* alongside the boxplot show the mean and standard deviation (SD). *Case* *1*: Identification of stent thrombosis mechanism in a haemodynamically stable STEMI patient (Appropriate, Mean = 8; SD ± 0.68). *Case* *2*: Re-evaluation with OCT after STEMI of a hazy non-culprit lesion which was initially treated conservatively (May be appropriate, Mean = 6; SD ± 1.94). *Case* *3*: Evaluation of mechanism in recurrent STEMI due to stent thrombosis in the proximal LAD (Appropriate, Mean = 9; SD ± 0.69). (*LAD* left anterior descending coronary artery, *OCT* optical coherence tomography, *SD* standard deviation, *STEMI* ST-elevation myocardial infarction)

OCT was considered ‘Appropriate’, in 26 scenarios (53%); (including stent thrombosis in STEMI, PCI in critical or distal left main coronary artery (LMCA) lesion, stent apposition in bioresorbable vascular scaffold (BVS)) (Tab. 4). OCT was considered ‘May be appropriate’, in 18 scenarios (37%). In five scenarios (10%), OCT was considered ‘Rarely appropriate’; (including late evaluation strut coverage, post FFR, proximal LMCA, pre rotablation, in stenosis of graft anastomosis). OCT was rated ‘Appropriate’ unanimously for identification of stent thrombosis mechanism.

### Remarks panellists

For ratings other than ‘Appropriate’, additional explanations were provided.

With regards to OCT-guided PCI of the LMCA, discrepancy existed between proximal (Rarely appropriate) and distal lesions (Appropriate). In the LMCA, proximal left anterior descending coronary artery (LAD) and bypass anastomosis, OCT was considered inferior to FFR and IVUS, due to insufficient contrast load in the ostium.

Inter-panellist appropriateness scores varied the most for OCT used to evaluate early or late discontinuation of dual antiplatelet therapy (DAPT) in relation to strut coverage, especially in BVS (Tab. 4, Case 6).

Although OCT adjacent to FFR was scored ‘Rarely appropriate’, assessing characteristics of thin-cap fibroatheroma was considered appropriate in a trial setting. Lack of medical evidence to support the use of OCT in the identification of early allograft vasculopathy or chronic total occlusion was mentioned as an explanation for deviating scores.

### Outliers

Outlier testing identified 19 outliers in 12 cases. One outlier was caused by a misunderstanding of the clinical scenario. Two outliers could be explained by preference of other imaging modalities over OCT. Other outliers could be explained by the lack of evidence or by the lack of clinical consequences. Because of the low rating sample, outliers were not discarded.

## Discussion

The increasing use of OCT contrasts with the scarce literature on OCT. Appropriate use criteria outline patient subgroups where the current medical evidence accompanied by expert opinion are combined to evaluate whether potential benefits exceed negative consequences of the treatment strategy in particular clinical scenarios.

Importantly, this is the first appropriate use criteria document on OCT in order to guide clinicians on the reasonable and appropriate use of OCT, namely preventing either under- or over-utilisation.

### Clinical scenarios

The clinical scenarios represented in this document cover a range of scenarios as encountered in clinical practice, with the purpose to cover actual and essential clinical situations.

Elaboration on only the noteworthy results of appropriate use criteria, e. g. outliers, unanimous ratings, discrepant results, will be described because depicting 49 clinical scenarios is too comprehensive.

OCT evaluation of stent thrombosis mechanism in haemodynamically stable STEMI patients was unanimously considered ‘Appropriate’, in line with current literature [6, 7]. Stent thrombosis is a therapeutic challenge for which treatment guideline recommendations have yet to be formulated by international societies. The pathophysiology of stent thrombosis, however, has been studied in several recent OCT studies, including PRESTIGE [7], PESTO [8], and the study by Taniwaki et al. [9]. The underlying mechanisms of stent thrombosis vary depending on the time elapsed after a PCI, and can be categorised into two classes; acute or late stent thrombosis. Leading causes of acute stent thrombosis are under-expansion and stent malapposition, whereas neo-atherosclerosis, besides malapposition, has been shown to be a major contributor to late stent thrombosis [7–9]. The PESTO study revealed that factors contributing to both categories of stent thrombosis can be identified with OCT in most cases [8].

Based on these data, it could be recommended to routinely use OCT to verify stent apposition. This seems especially appropriate in proximal segments, such as the LMCA or proximal LAD, where stent thrombosis would have major consequences due to the large myocardium at risk. Since the incidence of stent malapposition is higher after complex procedures at bifurcations, intravascular imaging is encouraged in complex bifurcation procedures in an LMCA or LAD/diagonal branch lesion [10]. In case of acute PCI with presence of stent thrombosis and haemodynamic stability, OCT can easily be performed [11].

In agreement with the literature, the use of OCT guidance in PCI in a critical or distal LMCA lesion was judged to be ‘Appropriate’. Critical lesions such as bifurcation lesions are a complex subgroup encountered in 15–20% of all PCIs. Compared with simple lesions, they have been associated with longer procedures and lower procedural success rates, i. e. less optimal angiographic and clinical outcomes [12, 13].

LMCA bifurcation lesions are an increasingly common site of complex stent implantation. Using OCT, Burzotta et al. were able to evaluate LMCA bifurcation lesions with a high degree of accuracy [14].

This approach is endorsed by the European Bifurcation Club, which recommends OCT guidance as well as IVUS guidance for the management of LMCA bifurcations [15]. The multicentre, randomised OPUS-CLASS study compared OCT versus IVUS in measuring of the lumen and in guidance in PCI. This study showed that OCT allows accurate and reproducible measurements of the coronary dimensions in day-to-day clinical practice [16], and is considerably more sensitive than IVUS in the detection of various indicators of suboptimal post-PCI lesion morphology (e. g. intra-stent tissue protrusion, incomplete stent apposition, stent edge dissection, and intra-stent thrombus) [17, 18].

In the current study, evaluating stent apposition in bioresorbable vascular scaffolds (BVS) was deemed ‘Appropriate’. The implant of BVS stents is associated with a higher risk of subacute stent thrombosis than the implant of drug-eluting stents (DES) [19]. There is a 3-fold higher incidence of acute and subacute BVS thrombosis, usually clustered within 30 days, than DES thrombosis [20–22]. The underlying mechanisms of stent thrombosis in BVS and DES are similar and include incomplete lesion coverage and under-expansion and malapposition of the stent [23]. The higher risk of stent thrombosis associated with BVS is probably due to thicker struts, which makes BVS less tolerant for suboptimal implantation.

In divergent clinical scenarios in which scientific evidence for the use of OCT is lacking, the use of OCT was considered ‘May be Appropriate’. The biggest dispersion in ratings with regards to the use of OCT was related to the evaluation of strut coverage of BVS. Neo-intimal coverage and endothelialisation after BVS implantation or its clinical time course has not been fully elucidated. The uncertainty which percentage of coverage is needed to safely stop DAPT may explain the experts’ doubts on the usefulness of OCT in this scenario [22].

The AIDA study showed that definite or probable device thrombosis occurs more often in BVS than in DES [22]. As a consequence, the Netherlands Society of Cardiology (NVVC) has adopted a consensus to maintain DAPT for 3 years if the Absorb BVS has been used, after which time the scaffold will most likely be completely absorbed.

OCT guidance could be of use in PCI procedural planning, for instance in stent sizing. In the ILUMIEN I study, OCT guidance changed the planned treatment strategy in 57% of cases. Remarkably, in 31% of cases, OCT led to stenting with a smaller diameter stent size [24]. Additionally, OCT led to changes in selection of stent length in 68%. In the setting of multiple complex lesions or a bifurcation lesion, the expert panel considered OCT guidance as ‘May be appropriate’. In contrast, in non-complex lesions angiography alone was considered sufficient for stent sizing, and the use of OCT was deemed ‘Rarely appropriate’.

Additionally, OCT-guided evaluation of late strut coverage in order to discontinue DAPT in patients with a high risk for bleeding was also considered ‘Rarely appropriate’. The panellists argued that with third generation DES and with sufficient stent apposition in the index procedure, DAPT could be stopped. The current literature endorses this point of view, and suggests that after implantation of new-generation DES, treatment with DAPT for 3–6 months may suffice to prevent stent thrombosis [25].

Another clinical scenario in which the use of OCT was considered ‘Rarely appropriate’ was the use of OCT adjacent to FFR. If the severity of the stenosis is uncertain, current practice guidelines propose the use of FFR. Nevertheless, the measurement of FFR may be unreliable for multiple stenoses, e. g. distal flow-limiting stenosis or collateral flow. Thus, minimal luminal area on OCT may be considered in these cases as an alternative. OCT is a suitable technique in such cases, except for ostial LMCA lesions [14]. Moreover, the DOCTORS study found that OCT-guided PCI modified the procedural strategy chosen by the physician in 50% of cases. OCT-guided PCI was associated with a post-procedural FFR >0.90 in 82.5% of patients, versus 64.2% of patients who underwent angiography-guided revascularisation [26].

OCT assessment of the LMCA is feasible and safe, and compared with IVUS more sensitive in detecting malapposition and edge dissection, and equivalent in the assessment of lumen and stent dimensions. However, direct comparisons with IVUS reveal that OCT achieves imaging completeness less often [27]. As a result, the use of OCT in the proximal LMCA was considered ‘Rarely appropriate’ by the panellists.

### Interpretation

The appropriate use criteria procedure is intended to be transparent for readers. Accordingly, the panellist’s numerical scores can be found online; Appendix Fig. 1, supplemental Appendix Figs. 2–14.

Because the division of the appropriateness scores in 3 categories may be arbitrary, scores should actually be viewed as a continuum. Nevertheless, the categories are proposed for clinical application. The array in clinical opinions for specific scenarios has been acknowledged. Thus the criteria can inform procedural use of OCT but physician judgement is required for patient-specific decisions. Additionally, this clinical scenario series is intended to be thorough, without being extensive. Therefore, some encountered clinical situations may not fit exactly into any of the scenarios presented, making certain procedures that are rated ‘Rarely appropriate’ admissible in particular settings. It is advised to clearly document these exemptions.

We envision that the interpretation and application of these criteria will provide insights into the way of care and will help to inform future guidelines for the use of OCT.

### Limitations

The validity of the observations may be influenced by the fact that 1) authors of the clinical scenarios also participated in the consensus panel and 2) there was no explicit balance in the panel between non-experts and experts since members of the Dutch Working Group on Interventional Cardiology participating in the expert panel most probably were more engaged and experienced in the OCT technology.

Although appropriate use of IVUS was not assessed routinely for the different clinical scenarios, it was taken in consideration during rating of the clinical scenarios. While these appropriate use criteria ratings reflect the current evidence accompanied by expert consensus, inevitably more research is needed to further identify not only when to use OCT but also when to choose OCT over other imaging modalities.

## Conclusion

In summary, this document presents, for the first time, side-by-side ratings by clinical experts of OCT in 49 clinical scenarios. OCT was considered ‘Appropriate’ when applied for guidance in PCI of the LMCA and the proximal LAD, evaluation of stent thrombosis in STEMI patients or apposition of BVS. OCT was considered as ‘May be appropriate’ when applied for evaluation of routine apposition or stent sizing. The use of OCT next to FFR was considered ‘Rarely appropriate’, unless applied in a trial setting. Additionally, the use of OCT for evaluation of strut coverage, bypass anastomoses or an ostium of the LMCA was considered ‘Rarely appropriate’.

## Caption Electronic Supplementary Material

Figures 2–14

## References

[1] Tearney GJ, Regar E, Akasaka T (2012). Consensus standards for acquisition, measurement, and reporting of intravascular optical coherence tomography studies: A report from the International Working Group for Intravascular Optical Coherence Tomography Standardization and Validation. J Am Coll Cardiol.

[2] Prati F, Regar E, Mintz GS (2010). Expert review document on methodology, terminology, and clinical applications of optical coherence tomography: Physical principles, methodology of image acquisition, and clinical application for assessment of coronary arteries and atherosclerosis. Eur Heart J.

[3] Prati F, Guagliumi G, Mintz GS (2012). Expert review document part 2: Methodology, terminology and clinical applications of optical coherence tomography for the assessment of interventional procedures. Eur Heart J.

[4] Hendel RC, Patel MR, Allen JM (2013). Appropriate use of cardiovascular technology: 2013 ACCF appropriate use criteria methodology update: A report of the American College of Cardiology Foundation Appropriate Use Criteria Task Force. J Am Coll Cardiol.

[5] Kolh P, Windecker S, Alfonso F (2014). ESC/EACTS Guidelines on myocardial revascularization. Eur J Cardiothorac Surg.

[6] Iannaccone M, Vadalà P, D’ascenzo F (2016). Clinical perspective of optical coherence tomography and intravascular ultrasound in STEMI patients. J Thorac Dis.

[7] Adriaenssens T, Joner M, Godschalk TC (2017). Optical Coherence Tomography Findings in Patients With Coronary Stent Thrombosis, a report of the PRESTIGE Consortium. Circulation.

[8] Souteyrand G, Amabile N, Mangin L (2016). PESTO Investigators. Mechanisms of stent thrombosis analysed by OCT: Insights from the national PESTO French registry. Eur Heart J.

[9] Taniwaki M, Radu MD, Zaugg S (2016). Mechanisms of very late drug-eluting stent thrombosis assessed by optical coherence tomography. Circulation.

[10] Tyczynski P, Ferrante G, Moreno-Ambroj C (2010). Simple versus complex approaches to treating coronary bifurcation lesions: Direct assessment of stent strut apposition by optical coherence tomography. Rev Esp Cardiol.

[11] Aoki J, Lansky AJ, Mehran R (2009). Early stent thrombosis in patients with acute coronary syndromes treated with drug-eluting and bare metal stents: The Acute Catheterization and Urgent Intervention Triage Strategy trial. Circulation.

[12] Iakovou I, Foin N, Andreou A, Viceconte N, Di Mario C (2011). New strategies in the treatment of coronary bifurcations. Herz.

[13] Latib A, Colombo A (2008). Bifurcation disease: What do we know, what should we do?. Jacc Cardiovasc Interv.

[14] Burzotta F, Dato I, Trani C (2015). Frequency domain optical coherence tomography to assess non-ostial left main coronary artery. EuroInt.

[15] Lassen JF, Holm NR, Stankovic G (2015). Percutaneous coronary intervention for coronary bifurcation disease: Consensus from the first 10 years of the European Bifurcation Club meetings. EuroIntervention.

[16] Tamburino C, Latib A, van Geuns RJ (2015). Contemporary practice and technical aspects in coronary intervention with bioresorbable scaffolds: A European perspective. EuroIntervention.

[17] Kubo T, Akasaka T, Shite J (2013). OCT compared with IVUS in a coronary lesion assessment: The OPUS-CLASS Study. Jacc Cardiovasc Imaging.

[18] Kawamori H, Shite J, Shinke T (2010). The ability of optical coherence tomography to monitor percutaneous coronary intervention: Detailed comparison with intravascular ultrasound. J Invasive Cardiol.

[19] Cassese S, Byrne RA, Ndrepepa G (2016). Everolimus-eluting bioresorbable vascular scaffolds versus everolimus-eluting metallic stents: A meta-analysis of randomised controlled trials. Lancet.

[20] Capodanno D, Gori T, Nef H (2015). Percutaneous coronary intervention with everolimus-eluting bioresorbable vascular scaffolds in routine clinical practice: Early and midterm outcomes from the European multicenter GHOST-EU registry. EuroIntervention.

[21] Serruys PW, Chevalier B, Sotomi Y (2016). Comparison of an everolimus-eluting bioresorbable scaffold with an everolimus-eluting metallic stent for the treatment of coronary artery stenosis (ABSORB II): A 3 year, randomised, controlled, single-blind, multicentre clinical trial. Lancet.

[22] Wykrzykowska JJ, Kraak RP, Hofma SH (2017). Bioresorbable scaffolds versus metallic Stents in routine PCI. N Engl J Med.

[23] Karanasos A, Van Mieghem N, van Ditzhuijzen N (2015). Angiographic and optical coherence tomography insights into bioresorbable scaffold thrombosis: Single-centre experience. Circ Cardiovasc Interv.

[24] Wijns W, Shite J, Jones MR (2015). Optical coherence tomography imaging during percutaneous coronary intervention impacts physician decision-making: ILUMIEN I study. Eur Heart J.

[25] Bitll JA, Baber U, Bradley SM (2016). Duration of dual Antiplatelet therapy: A systematic review for the 2016 ACC/AHA guideline focused update on duration of dual Antiplatelet therapy in patients with coronary artery disease. J Am Coll Cardiol.

[26] Meneveau N, Souteyrand G, Motreff P (2016). Optical coherence Tomography to optimize results of Percutaneous coronary intervention in patients with non-ST-elevation acute coronary syndrome. Circulation.

[27] Fujino Y, Bezerra HG, Attizzani GF (2013). Frequency-domain optical coherence tomography assessment of unprotected left main coronary artery disease—a comparison with intravascular ultrasound. Catheter Cardiovasc Interv.

