# Supplementary material for: Appropriate use criteria for optical coherence tomography guidance in percutaneous coronary interventions: Recommendations of the working group of interventional cardiology of the Netherlands Society of Cardiology
Source: Neth Heart J. 2018 Aug 31;26(10):473–83. doi: 10.1007/s12471-018-1143-z (PMC6150879; doi:10.1007/s12471-018-1143-z)
Supplement: Supplementary file 1 — Figures 2–14 [file 12471_2018_1143_MOESM1_ESM.docx]

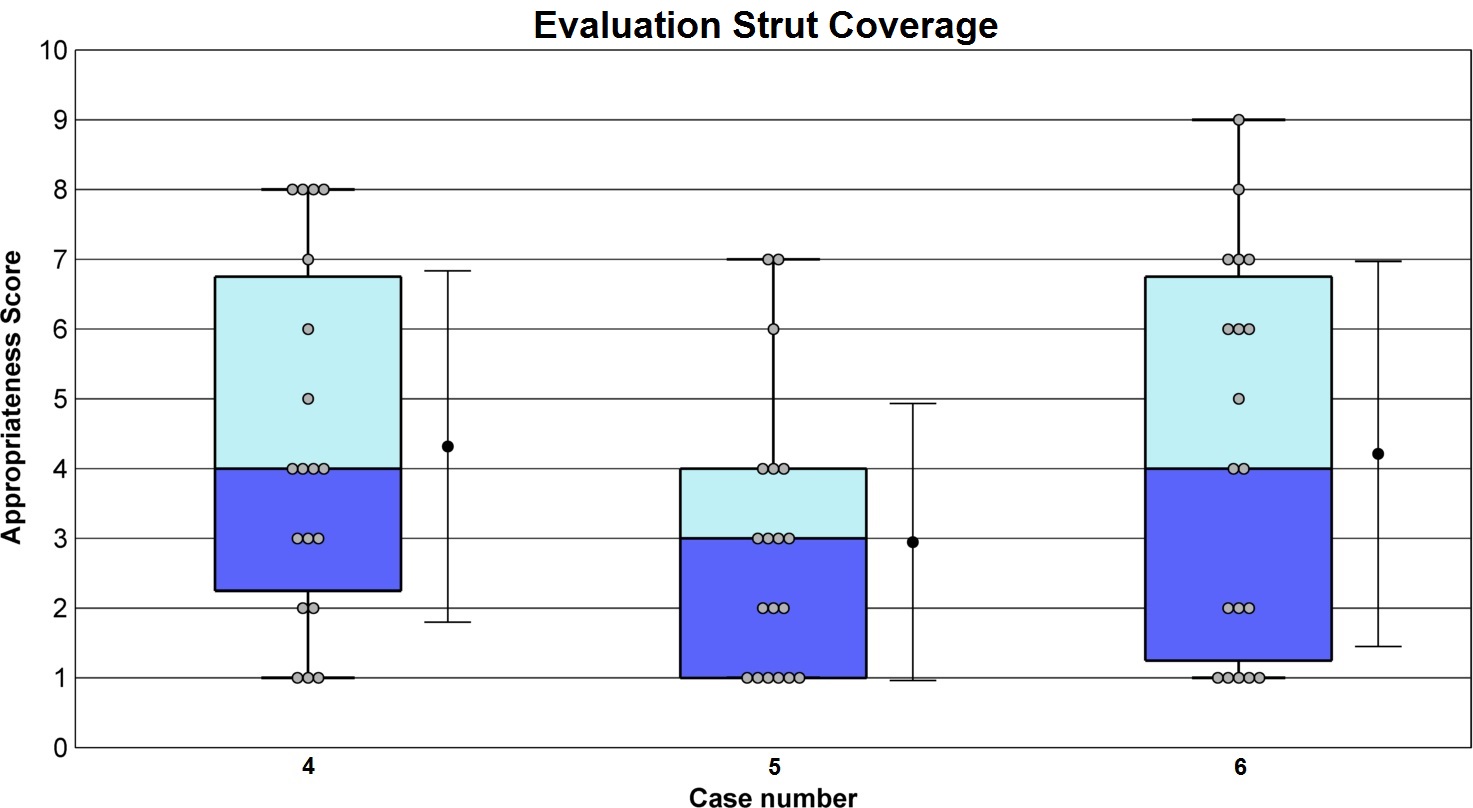


**Fig. 2** OCT appropriate use criteria scores for evaluation strut coverage

On each box, the central mark indicates the median, and the bottom and top edges of the box indicate the 25th and 75th percentiles, respectively. The whiskers extend to the most extreme data points not considered outliers, and the outliers are plotted individually as a red dot. The grey dots represent the individual scores of the panellists. The whiskers alongside the boxplot show the mean and standard deviation (SD).

Case 4. Evaluation of strut coverage 4 weeks after initial stent placement in a patient with a high bleeding risk (discontinuing DAPT). (May be appropriate, Mean=4; SD±2.51)

Case 5. Evaluation of strut coverage 12 weeks after initial stent placement in a patient with a high bleeding risk who requires surgery (discontinuing DAPT). (Rarely appropriate, Mean=3; SD±1.98)

Case 6. Evaluation of BVS after ~1.5 years for discontinuing DAPT. (May be appropriate, Mean=4; SD±2.76)

*BVS* bioresorbable vascular scaffold, *DAPT* dual antiplatelet therapy, *OCT* optical coherence tomography, *SD* standard deviation


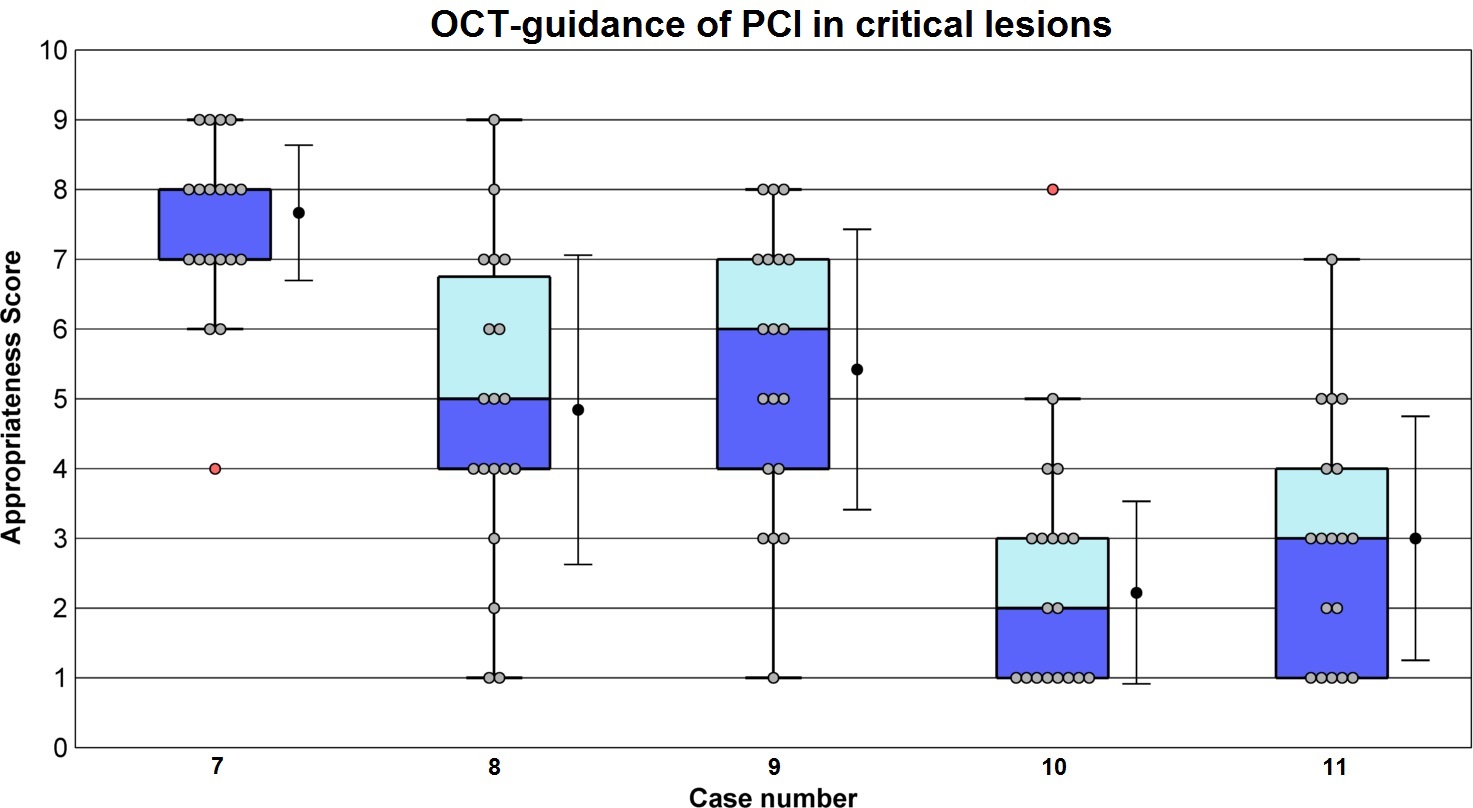


**Fig. 3** OCT appropriate use criteria scores for OCT guidance in PCI of critical lesions

On each box, the central mark indicates the median, and the bottom and top edges of the box indicate the 25th and 75th percentiles, respectively. The whiskers extend to the most extreme data points not considered outliers, and the outliers are plotted individually as a red dot. The grey dots represent the individual scores of the panellists. The whiskers alongside the boxplot show the mean and standard deviation (SD).

Case 7. Guiding in complicated PCI with unknown (ap)position of the stent in LM and post PCI with possible stent fracture after overexpansion. (Appropriate, Mean=8; SD±1.26)

Case 8. Guiding in PCI bifurcation lesion for sizing and stent strategy. (May be appropriate, Mean= 5; SD±2.21)

Case 9. Guiding in PCI to determine landing zone stent and stent length in angiographic diffuse long lesion. (May be appropriate, Mean=6; SD±2.00)

Case 10. OCT next to significant FFR for evaluation stenosis severity. (Rarely appropriate, Mean=2; SD±1.83)

Case 11. OCT next to non-significant FFR for evaluation stenosis severity. (Rarely appropriate, Mean=3; SD±1.74)

*FFR* fractional flow reserve*, OCT* optical coherence tomography, *PCI* percutaneous coronary intervention, *SD* standard deviation


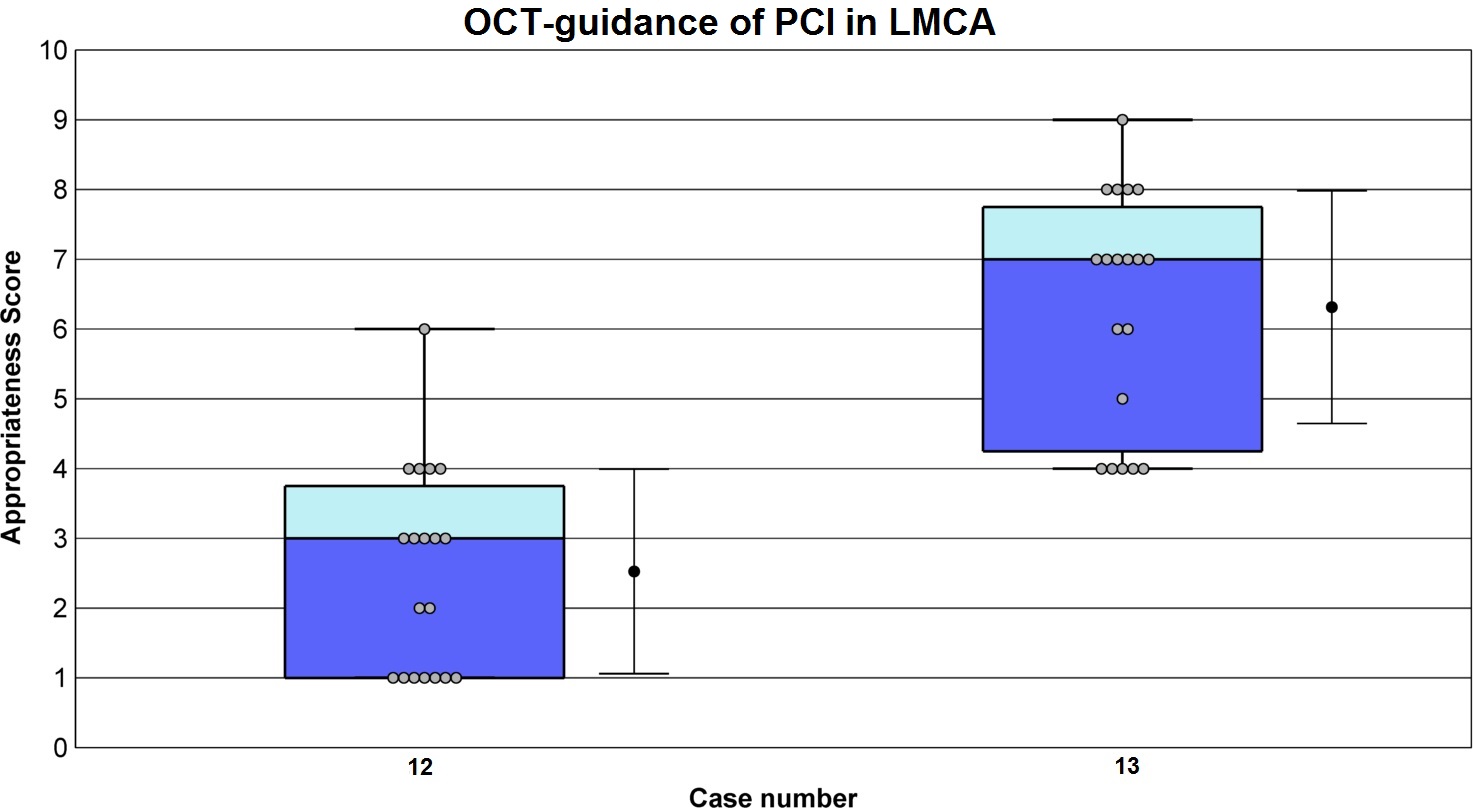


**Fig. 4** OCT appropriate use criteria scores for OCT guidance in PCI of LMCA

On each box, the central mark indicates the median, and the bottom and top edges of the box indicate the 25th and 75th percentiles, respectively. The whiskers extend to the most extreme data points not considered outliers, and the outliers are plotted individually as a red dot. The grey dots represent the individual scores of the panellists. The whiskers alongside the boxplot show the mean and standard deviation (SD).

Case 12. OCT guidance in PCI of the proximal LMCA. (Rarely appropriate, Mean=2; SD±1.46)

Case 13. OCT guidance in PCI of the distal LMCA. (Appropriate, Mean=7; SD±1.66)

*LMCA* left main coronary artery, *OCT* optical coherence tomography, *PCI* percutaneous coronary intervention, *SD* standard deviation


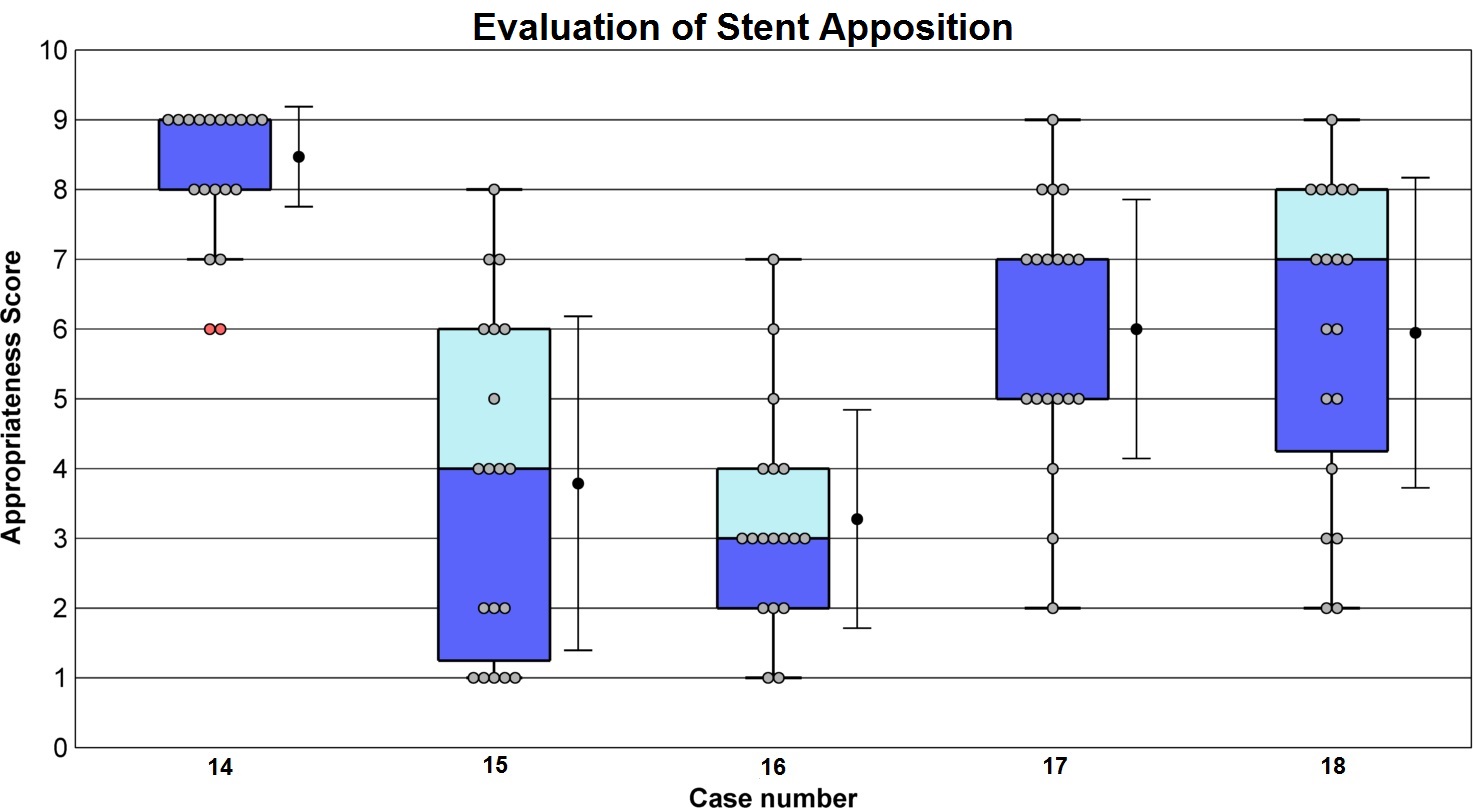


**Fig. 5** OCT appropriate use criteria scores for evaluation of stent apposition

On each box, the central mark indicates the median, and the bottom and top edges of the box indicate the 25th and 75th percentiles, respectively. The whiskers extend to the most extreme data points not considered outliers, and the outliers are plotted individually as a red dot. The grey dots represent the individual scores of the panellists. The whiskers alongside the boxplot show the mean and standard deviation (SD).

Case 14. Evaluating thrombosis mechanism in extensive stent thrombosis. (Appropriate, Mean=9; SD±1.03)

Case 15. Evaluating stent apposition post PCI in non-complex lesion. (May be appropriate, Mean=4; SD±2.39)

Case 16. Evaluating severe calcified lesion for treatment strategy (rotablator). (Rarely appropriate, Mean=3; SD±1.56)

Case 17. Evaluating stent apposition after rotablator treatment in complex diffuse long lesion and placement of multiple stents. (Appropriate, Mean=7; SD±1.85)

Case 18. Evaluating stent apposition after extensive post dilatation in an initially undersized stent. (Appropriate, Mean=7; SD±2.22)

*OCT* optical coherence tomography, *PCI* percutaneous coronary intervention, *SD* standard deviation


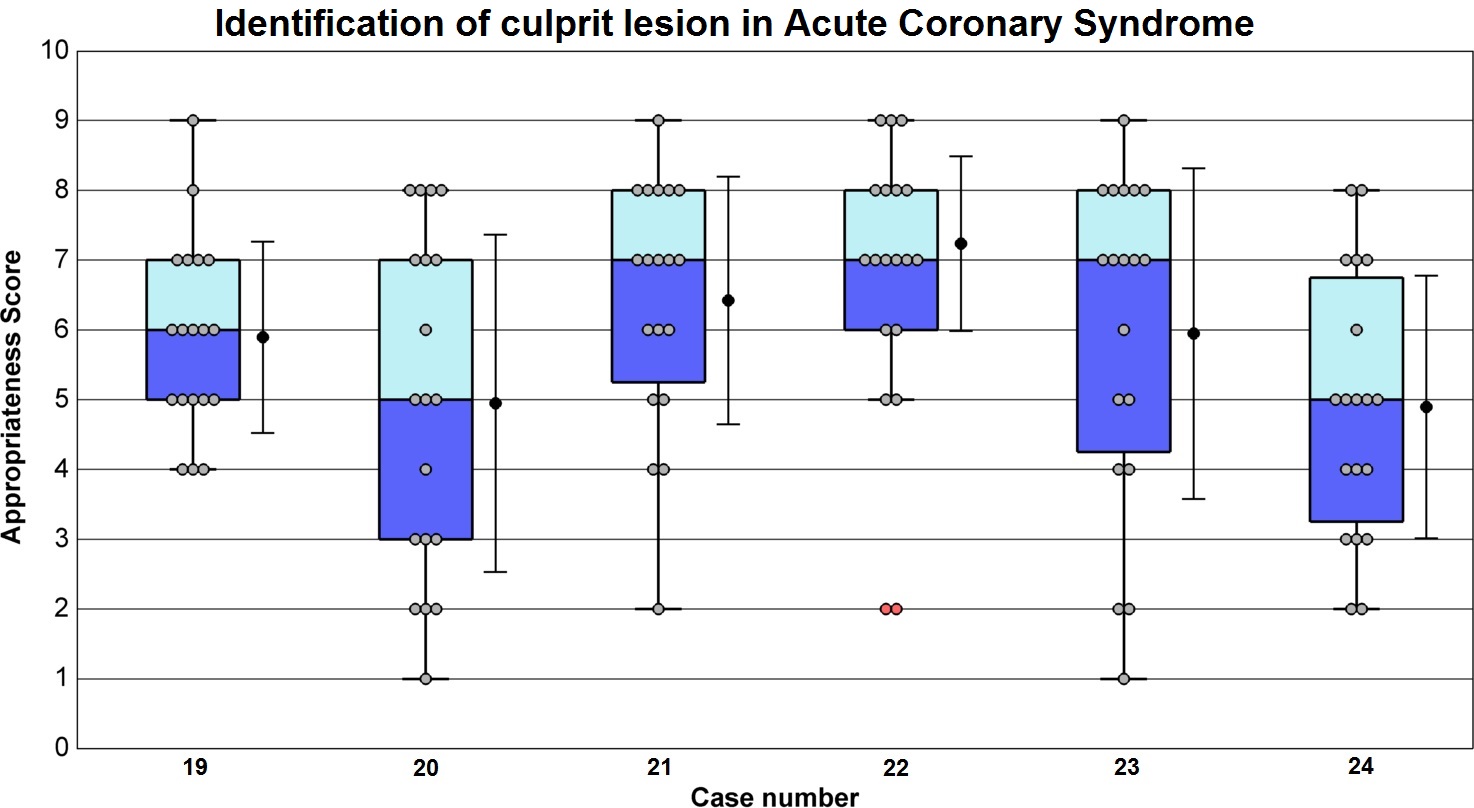
**Fig. 6** OCT appropriate use criteria scores for identification of culprit lesion in Acute Coronary Syndrome

On each box, the central mark indicates the median, and the bottom and top edges of the box indicate the 25th and 75th percentiles, respectively. The whiskers extend to the most extreme data points not considered outliers, and the outliers are plotted individually as a red dot. The grey dots represent the individual scores of the panellists. The whiskers alongside the boxplot show the mean and standard deviation (SD).

Case 19. Identification culprit lesion in NSTEMI with angiographic two significant stenosis and no decisive answer on which one is the culprit. (May be appropriate, Mean=6; SD±1.37)

Case 20. Identification mechanism NSTEMI (spasm vs. plaque rupture) after thrombectomy followed by severe spasm. (May be appropriate, Mean=5; SD±2.41)

Case 21. Identification culprit lesion in NSTEMI with abnormal ECG and angiographic no evident thrombus or occlusion. (Appropriate, Mean=7; SD±1.77)

Case 22. Identification plaque erosion. (Appropriate, Mean=7; SD±2.02)

Case 23. Identification culprit lesion in OHCA with angiographic signs (haziness) (Appropriate, Mean=7; SD±2.36)

Case 24. Identification culprit lesion in MI with abnormal ECG and angiographic intermediate stenosis. (May be appropriate, Mean=5; SD±1.88)

*ECG* electrocardiogram *MI* myocardial infarction *NSTEMI* non-ST-elevation myocardial infarction *OCT* optical coherence tomography, *OHCA* out-of-hospital cardiac arrest, *SD* standard deviation


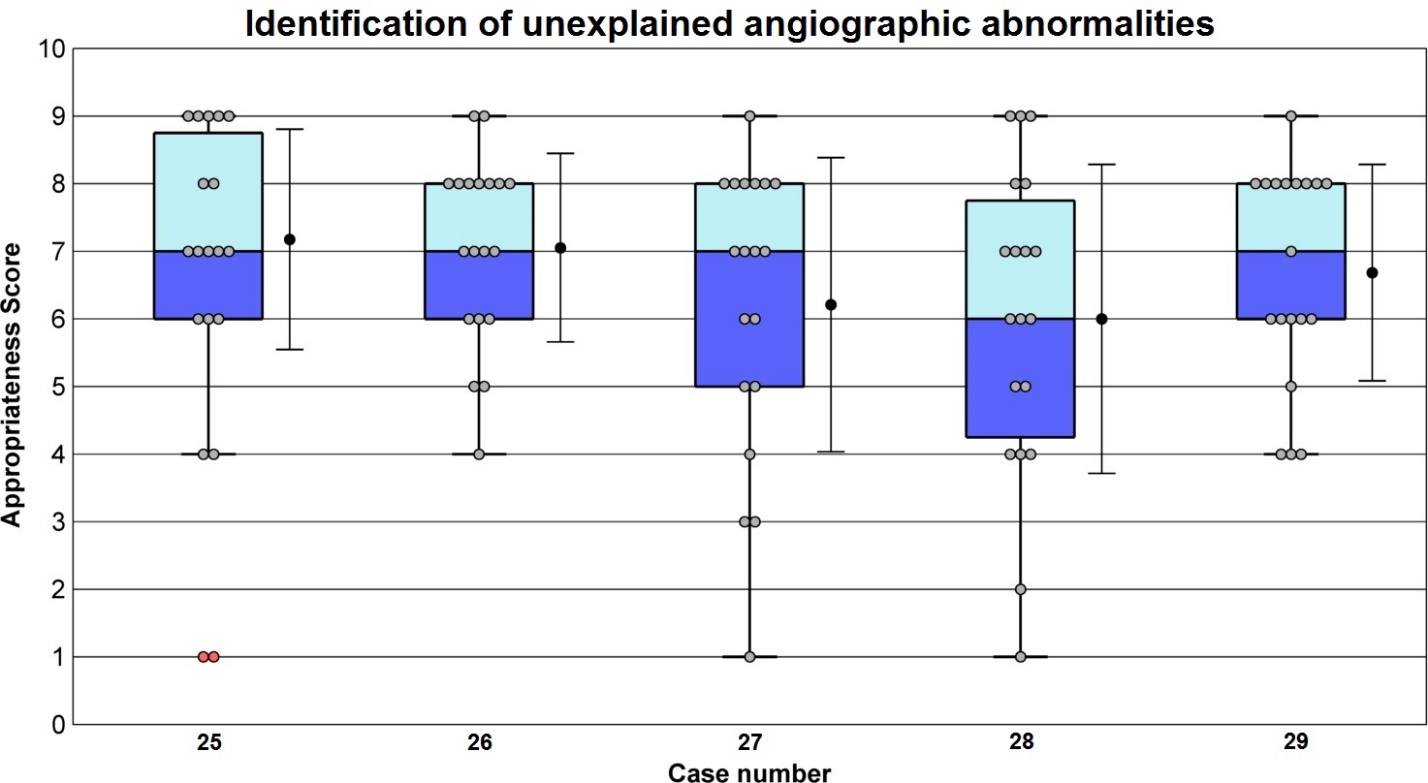


**Fig. 7** OCT appropriate use criteria scores for identification of unexplained angiographic abnormalities

On each box, the central mark indicates the median, and the bottom and top edges of the box indicate the 25th and 75th percentiles, respectively. The whiskers extend to the most extreme data points not considered outliers, and the outliers are plotted individually as a red dot. The grey dots represent the individual scores of the panellists. The whiskers alongside the boxplot show the mean and standard deviation (SD).

Case 25. Unravel mechanism for distal occlusion in coronary artery without proximal lesion (local problem or emboli?) (Appropriate, Mean=7; SD±2.48)

Case 26. Control OCT 5 days after initial angiography in NSTEMI patient which was treated conservatively. (Appropriate, Mean=7; SD±1.39)

Case 27. Evaluation haziness (thrombus) in proximal LAD in STEMI patient with incurable cancer (local problem of emboli?) (Appropriate, Mean=7; SD±2.17)

Case 28. Discrepancy between angiographic finding (intermediate stenosis) and FFR (borderline significant) (May be appropriate, Mean=6; SD±2.28)

Case 29. Evaluation angiographic haziness in transient STEMI (Appropriate, Mean=7; SD±1.60)

*FFR* fractional flow reserve *LAD* left anterior descending *NSTEMI* non-ST-elevation myocardial infarction *OCT* optical coherence tomography, *SD* standard deviation, *STEMI* ST-elevation myocardial infarction


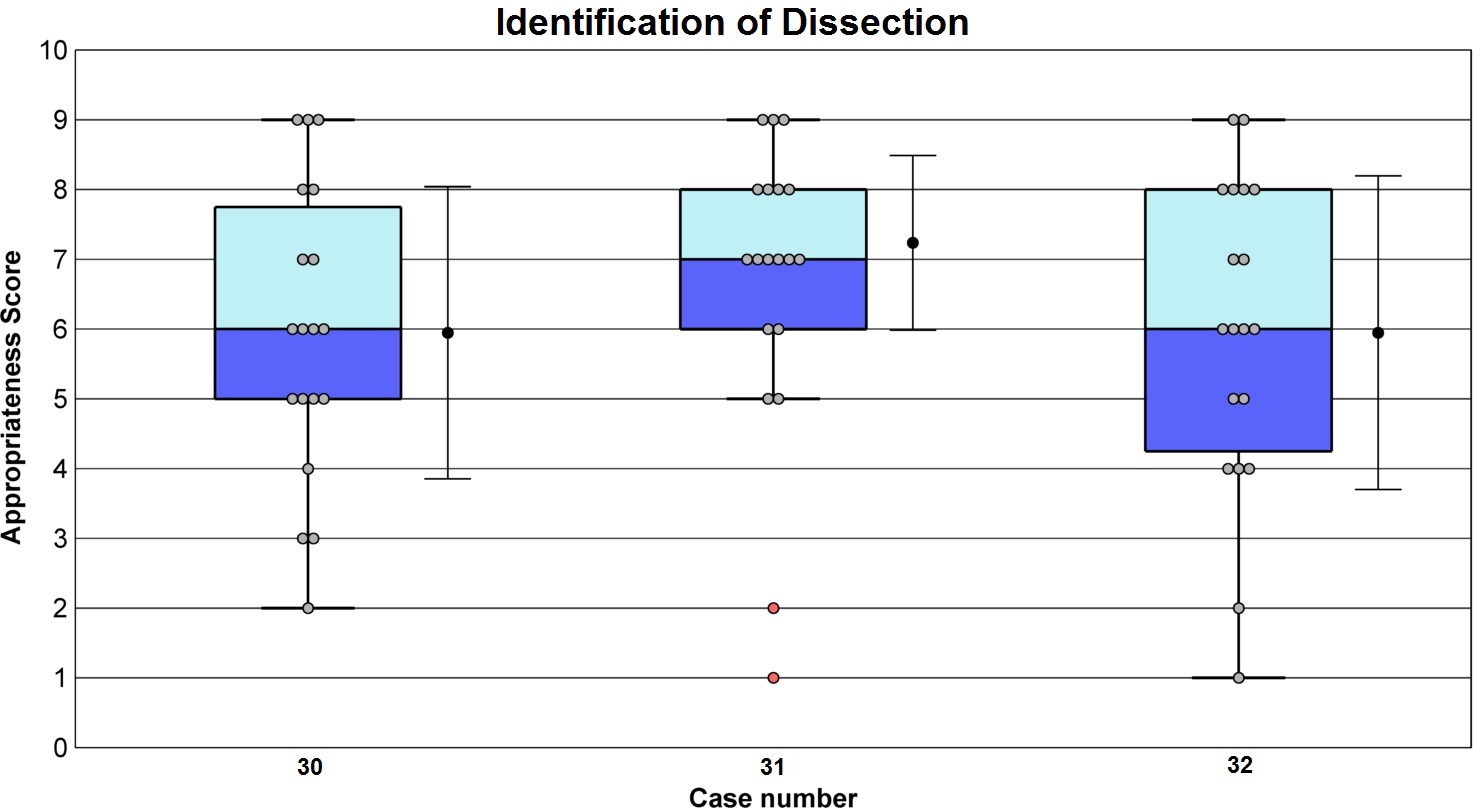


**Fig. 8** OCT appropriate use criteria scores for identification of dissection

On each box, the central mark indicates the median, and the bottom and top edges of the box indicate the 25th and 75th percentiles, respectively. The whiskers extend to the most extreme data points not considered outliers, and the outliers are plotted individually as a red dot. The grey dots represent the individual scores of the panellists. The whiskers alongside the boxplot show the mean and standard deviation (SD).

Case 30. Confirmation of SCAD in young patient without classical risk factors for atherosclerotic coronary artery disease. (May be appropriate, Mean=6; SD±2.09)

Case 31. Identification thrombosis mechanism after thrombosuction resulting in a normal angiography in a patient with a mechanical valve. (Appropriate, Mean=7; SD±2.16)

Case 32. Confirmation of SCAD in young patient with classical risk factors for atherosclerotic coronary disease. (May be appropriate, Mean=6; SD±2.24)

*OCT* optical coherence tomography, *SCAD* spontaneous coronary artery dissection *SD* standard deviation


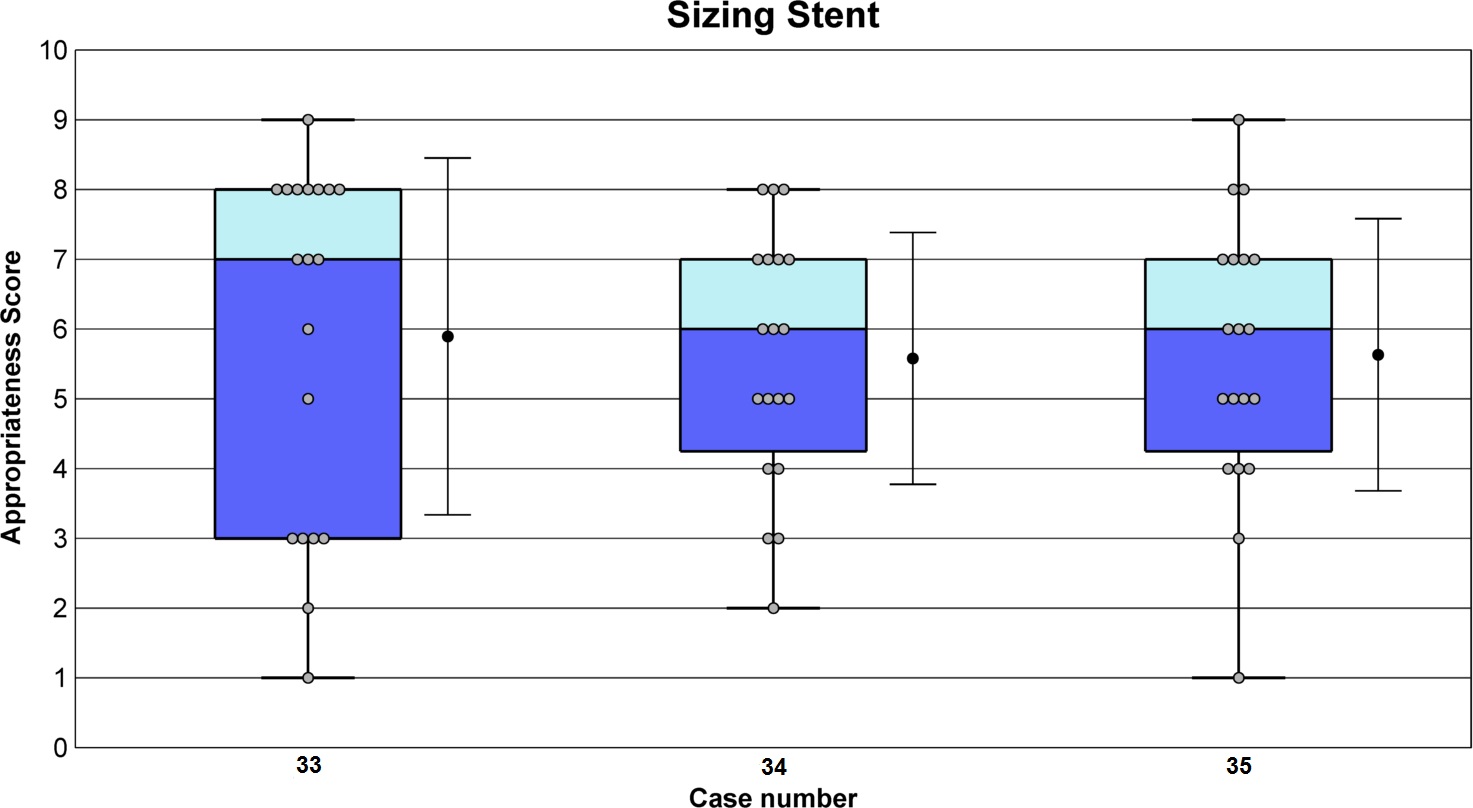


**Fig. 9** OCT appropriate use criteria scores for sizing stent

On each box, the central mark indicates the median, and the bottom and top edges of the box indicate the 25th and 75th percentiles, respectively. The whiskers extend to the most extreme data points not considered outliers, and the outliers are plotted individually as a red dot. The grey dots represent the individual scores of the panellists. The whiskers alongside the boxplot show the mean and standard deviation (SD).

Case 33. Sizing for covered stent with risk on blocking substantial side branch. (Appropriate, Mean=7; SD±2.55)

Case 34. Sizing for stent in hazy angiography with multiple complex lesions. (May be appropriate, Mean=6; SD±1.80)

Case 35. Stent sizing in bifurcation lesion (pre PCI). (May be appropriate, Mean=6; SD±1.94)

*OCT* optical coherence tomography, *PCI* percutaneous coronary intervention, *SD* standard deviation


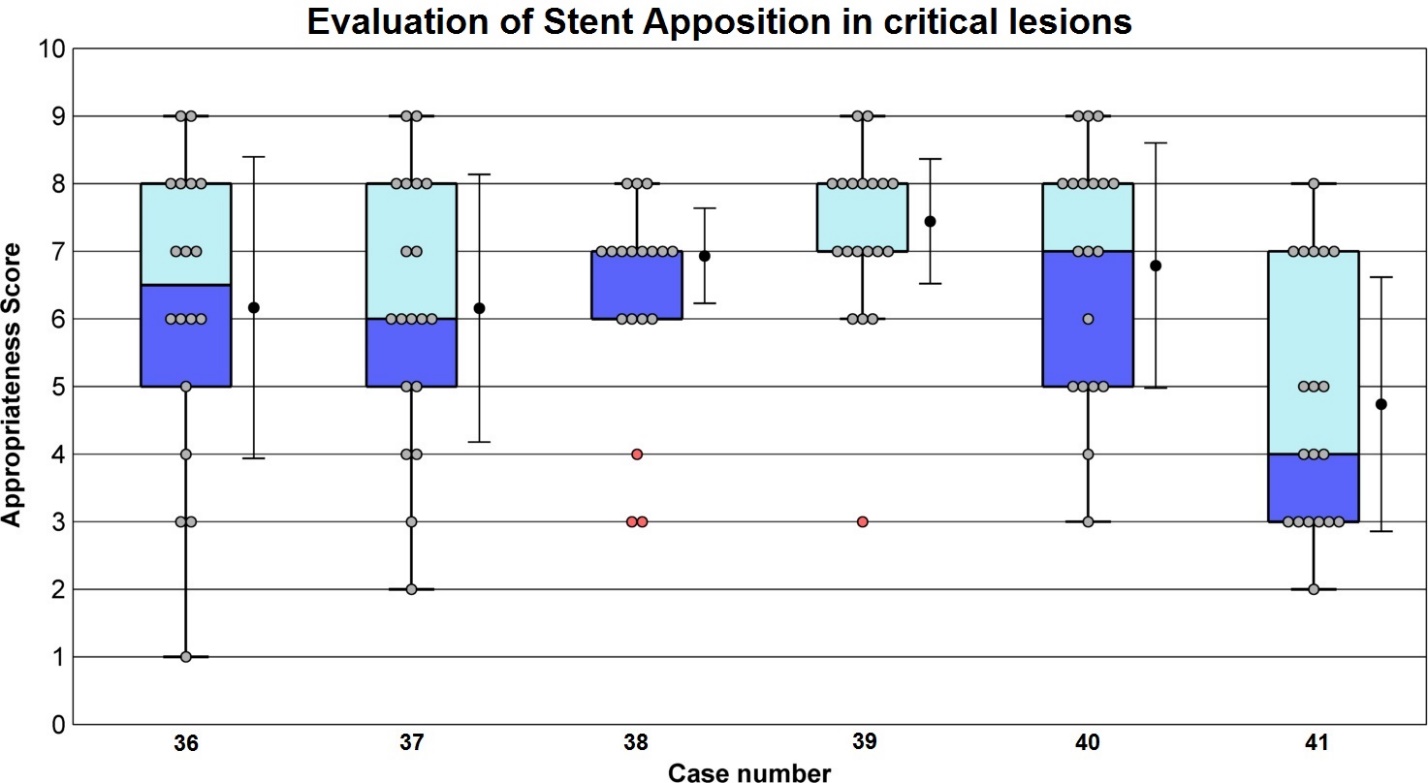


**Fig. 10** OCT appropriate use criteria scores for evaluation of stent apposition in critical lesions

On each box, the central mark indicates the median, and the bottom and top edges of the box indicate the 25th and 75th percentiles, respectively. The whiskers extend to the most extreme data points not considered outliers, and the outliers are plotted individually as a red dot. The grey dots represent the individual scores of the panellists. The whiskers alongside the boxplot show the mean and standard deviation (SD).

Case 36. Control OCT after 2 weeks to evaluate stent apposition in proximal LAD with suspected malapposition during initial angiography. (Appropriate, Mean=6.5; SD±2.22)

Case 37. Control OCT for stent apposition in a patient with a high bleeding risk and angiographically suspected under-expansion. (May be appropriate, Mean=6; SD±1.97)

Case 38. Evaluating stent apposition in bifurcation lesion (post PCI)

Case 39. Identification of the mechanism behind a distal occlusion in a coronary vessel with multiple mild plaques proximally (local or emboli of other origin?). (Appropriate, Mean=7; SD±1.35)

Case 40. Evaluating stent apposition in a patient with a high bleeding risk with the intention to keep the duration of DAPT treatment as short as possible. (Appropriate, Mean=7; SD±1.81)

Case 41. Routine use of OCT for evaluation stent apposition in PCI of proximal LAD. (May be appropriate, Mean=4; SD±1.88)

*DAPT* dual antiplatelet therapy *LAD* left anterior descending *OCT* optical coherence tomography, *PCI* percutaneous coronary intervention, *SD* standard deviation


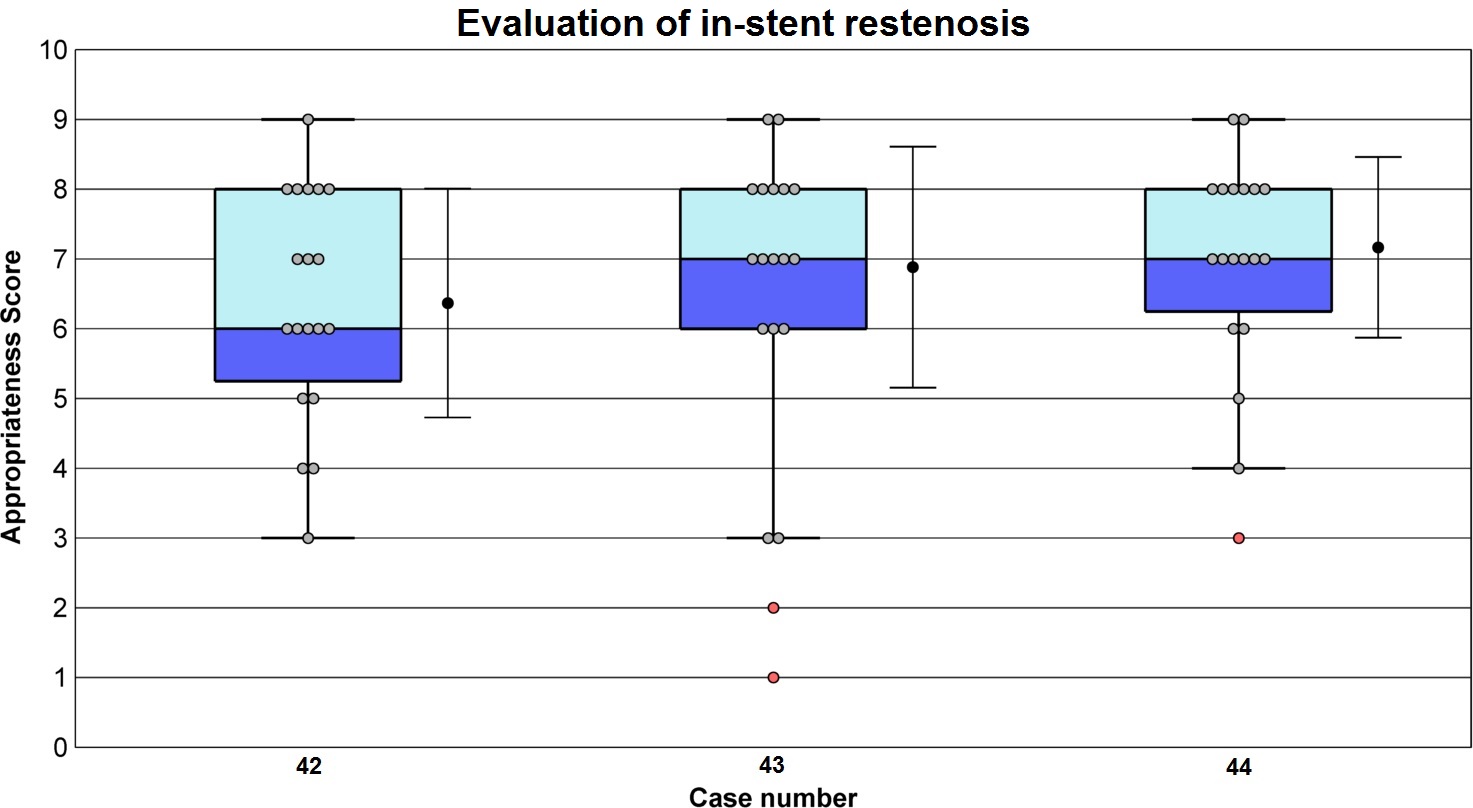


**Fig. 11** OCT appropriate use criteria scores for evaluation of in-stent restenosis

On each box, the central mark indicates the median, and the bottom and top edges of the box indicate the 25th and 75th percentiles, respectively. The whiskers extend to the most extreme data points not considered outliers, and the outliers are plotted individually as a red dot. The grey dots represent the individual scores of the panellists. The whiskers alongside the boxplot show the mean and standard deviation (SD).

Case 42. OCT identification of the mechanism of ISR in order to guide therapy, i.e. DES vs. DEB after 1st restenosis. (May be appropriate, Mean=6; SD±1.64)

Case 43. OCT identification of the mechanism of ISR in order to guide therapy, i.e. DES vs. DEB after 2nd restenosis. (Appropriate, Mean=7; SD±2.35)

Case 44. OCT identification of the mechanism of ISR in order to guide therapy, i.e. DES vs. DEB after 3rd restenosis. (Appropriate, Mean=7; SD±1.58)

*DEB* drug-eluting balloon *DES* drug eluting stent *ISR* in-stent restenosis *OCT* optical coherence tomography, *SD* standard deviation


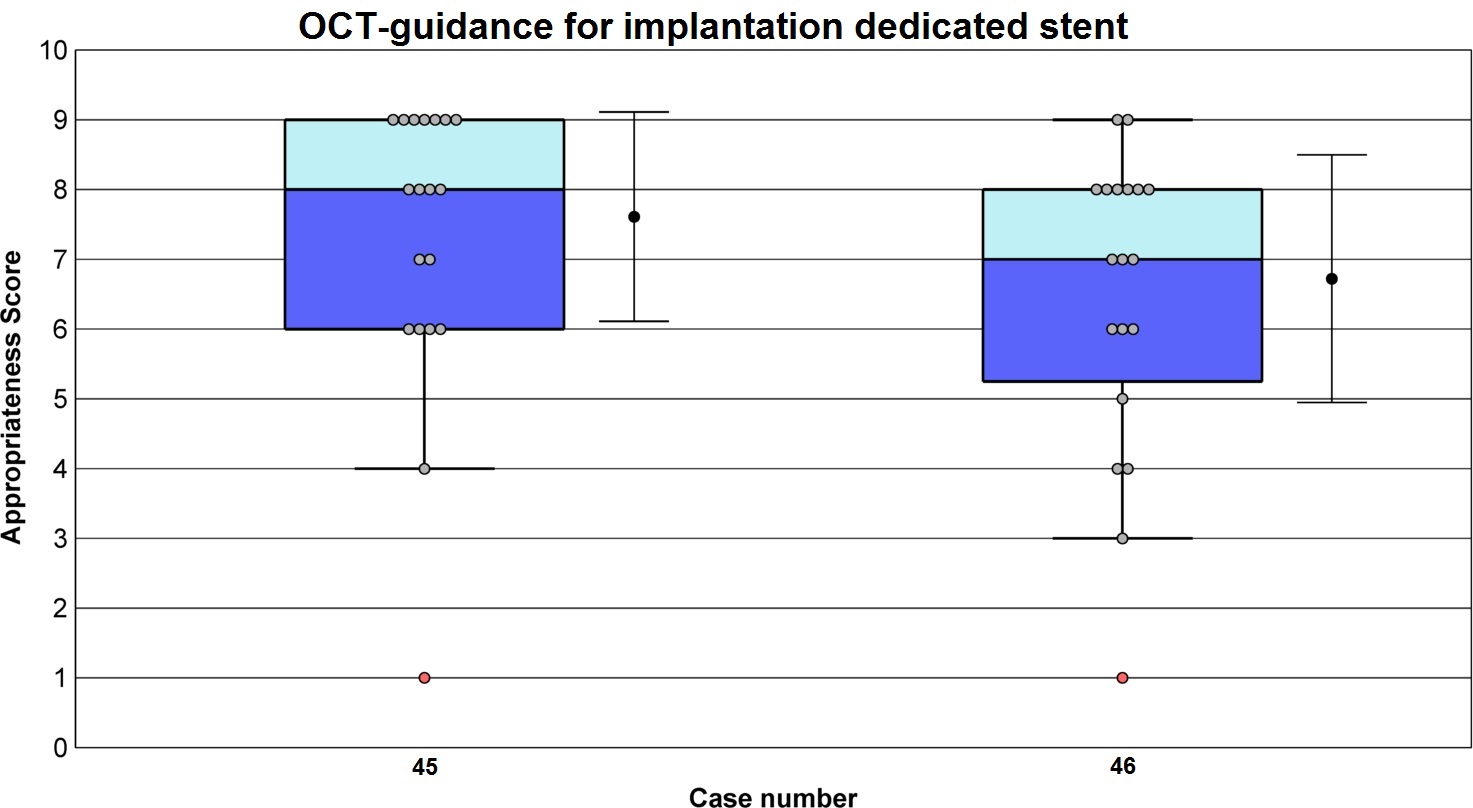


**Fig. 12** OCT appropriate use criteria scores for OCT guidance for implantation dedicated stent

On each box, the central mark indicates the median, and the bottom and top edges of the box indicate the 25th and 75th percentiles, respectively. The whiskers extend to the most extreme data points not considered outliers, and the outliers are plotted individually as a red dot. The grey dots represent the individual scores of the panellists. The whiskers alongside the boxplot show the mean and standard deviation (SD).

Case 45. Evaluation of stent apposition in a BVS. (Appropriate, Mean=8; SD±2.10)

Case 46. Evaluation of stent apposition in a self-expandable stent. (Appropriate, Mean=7; SD±2.16)

*BVS* bioresorbable vascular scaffold *OCT* optical coherence tomography, *SD* standard deviation


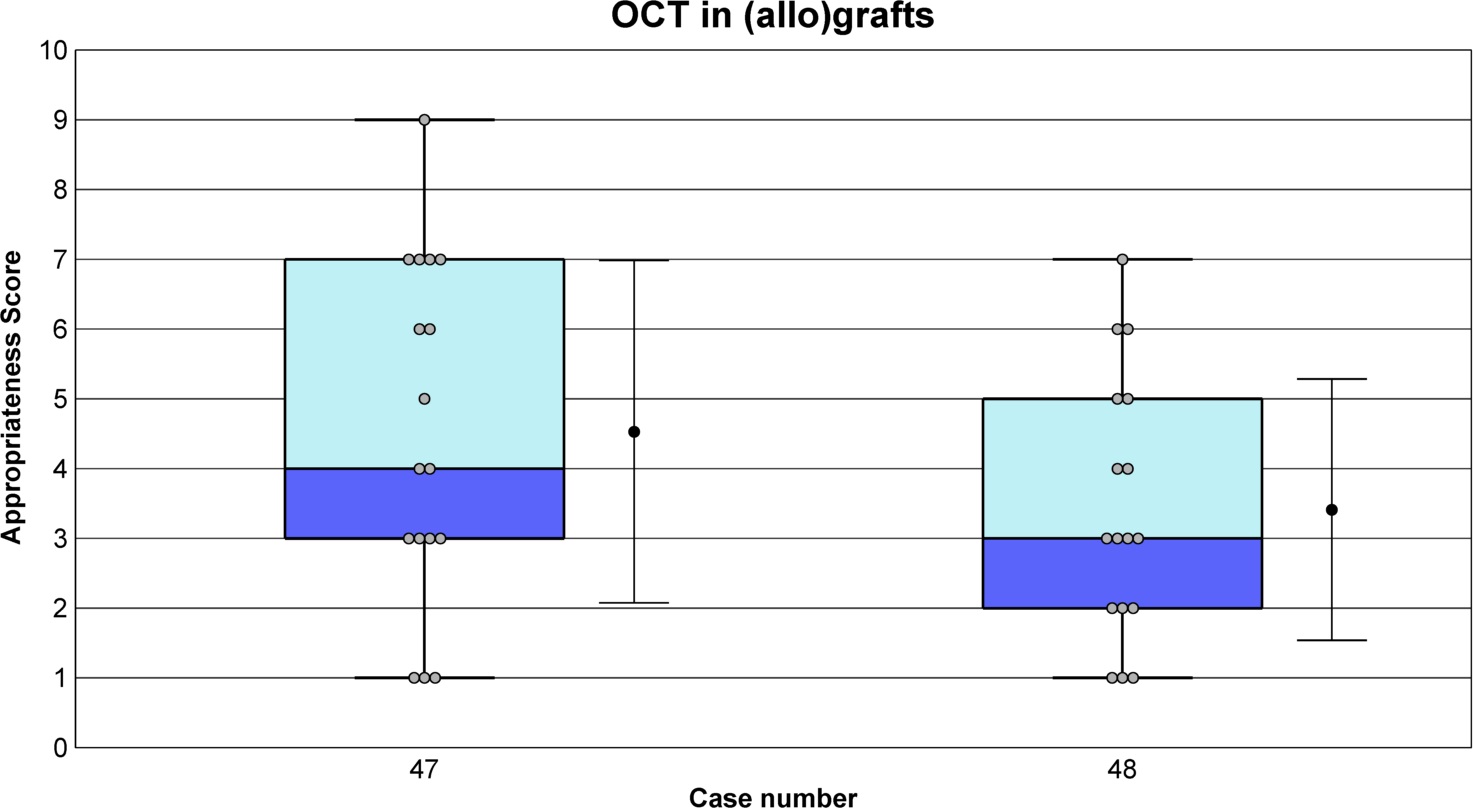


**Fig. 13** OCT appropriate use criteria scores for OCT in grafts

On each box, the central mark indicates the median, and the bottom and top edges of the box indicate the 25th and 75th percentiles, respectively. The whiskers extend to the most extreme data points not considered outliers, and the outliers are plotted individually as a red dot. The grey dots represent the individual scores of the panellists. The whiskers alongside the boxplot show the mean and standard deviation (SD).

Case 47. Detection of early cardiac allograft vasculopathy after heart transplant. (May be appropriate, Mean=4; SD±2.45)

Case 48. Detection of stenosis of a CABG anastomosis. (Rarely appropriate, Mean=3; SD±1.87)

*CABG* coronary artery bypass grafting *OCT* optical coherence tomography, *SD* standard deviation


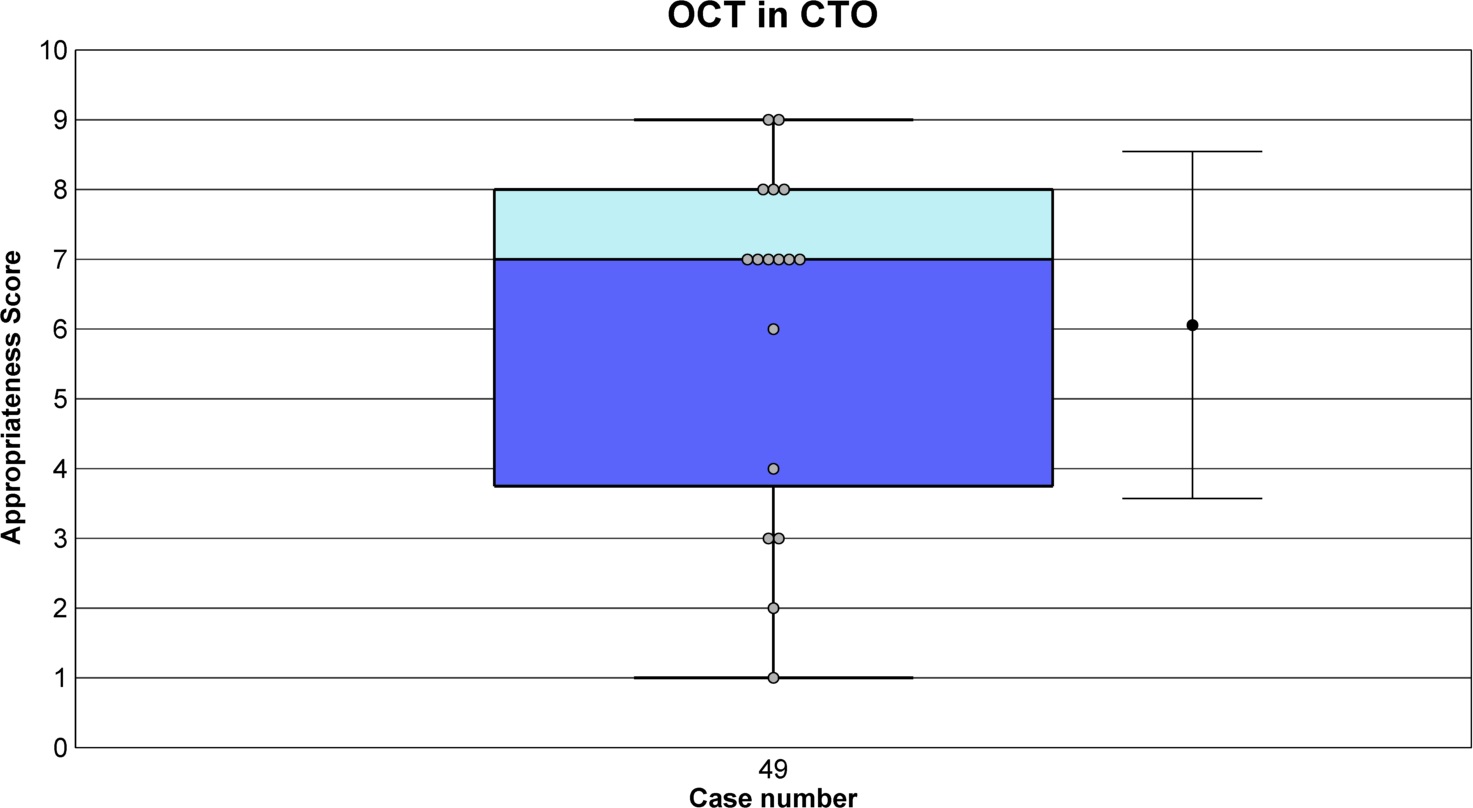


**Fig. 14** OCT appropriate use criteria scores for OCT in chronic total occlusion

On each box, the central mark indicates the median, and the bottom and top edges of the box indicate the 25th and 75th percentiles, respectively. The whiskers extend to the most extreme data points not considered outliers, and the outliers are plotted individually as a red dot. The grey dots represent the individual scores of the panellists. The whiskers alongside the boxplot show the mean and standard deviation (SD).

Case 49. Evaluation of multiple dissection-like images outside the stent in the sub-intimal path of a previous CTO during follow-up angiography after CTO recanalisation. (Appropriate, Mean=7; SD±2.48)

*CTO* chronic total occlusion *OCT* optical coherence tomography, *SD* standard deviation
